# The Texas Medical Law

**Published:** 1894-04

**Authors:** 


THE TEXAS MEDICAL LAW.—The Illinois State Board of Health in compiling a synopsis of the laws governing the practice in the several States, makes a bad break with regard to the law in Texas. We would like to know where Secretary Riley got his information; the old warhorse Sanitarian Rauch never made any such mistake; he was always sure he was right, and then went ahead. We only wish that the facts were as they are stated to be; for if there is any one thing that this JOURNAL has ventilated, it is the fact that in Texas we have, virtually, no law on the subject; and it has been a kind of standing subject for editorial lament that the profession have for years tried in vain to procure the passage of an act that would give us some protection against the hordes of pretenders who come into Texas to practice.

Secretary Riley surely has not been a reader of the TEXAS MEDICAL JOURNAL; that is no excuse however, for his ignorance on the subject. It is notorious that the Texas law requires nothing but a *diploma* (a license can be procured by virtue of it alone) and the clerk of the county court is the judge of that diploma, even though it be written in Latin, and he is unable to read it. In other words, in Texas "everything (in the way of a diploma) goes."

There is even less excuse for the ignorance of the New York *Medical Record*, the leading journal of America, on this subject, than for the Illinois Board; (and that is none at all, for when a board makes an official announcement, it should be sure its information is reliable). The editor of the *Record* reads; he reads the TEXAS MEDICAL JOURNAL, we know he does; it has been a kind of hair cloth undershirt to him, judging from certain wincing and kicks in times past, but the *Record* in commenting on the compilation of medical laws by the Illinois board does not correct the mistake, but shakes hands with himself in congratulation, and says: "It is encouraging in this connection to notice that there are sixteen States in which a diploma of itself is no license to practice, and in which an extra and independent State examination is demanded by law, before the applicant can be qualified." And Texas is classed with these. "Extra and independent State examinations" *nothing*: Texas has not even a State Board of Medical Examiners; and district boards only examine those who present themselves voluntarily, and who have no diploma. Contrary to all reason—contrary to Webster, who says a diploma is only an evidence of a degree having been conferred, and conveys no rights whatever; contrary to all authority, in Texas a diploma is authority to practice, and so recognized by its law; the conditions being that it shall be recorded. Very true, the law does require that the diploma shall be vised by a district board, and license procured from said district board,—but *without examination*. The latter part of the requirement—the appearance before a district board is usually dispensed with. The owner, (or it may be, the holder, only, of another man's diploma, so far as we know or have any means of ascertaining), simply registers the diploma in the office of the county clerk, and goes to practicing; and until recently it was generally thought that that was all that is required by the law. But the prosecution of the Kennedy case,—mention of which is made elsewhere,—and a full account of which will appear in our next, developed the fact that the registration is not all that is required, and that many of our best known physicians are not virtually legal practitioners, in that they have neglected to take out a license.

If the mistake in classification, and the currency given by the *Record* to the statement of the Illinois Board that Texas requires a State examination shall deter any of the unqualified from coming to Texas to practice, we will readily forgive both parties.

